# Hair transplantation versus scalp micropigmentation: A retrospective study of treatment selection in persons with female pattern hair loss

**DOI:** 10.1111/jocd.16429

**Published:** 2024-06-18

**Authors:** Jae Hyun Park, Narae Kim, Seung Hyun You

**Affiliations:** ^1^ Dana Plastic Surgery Clinic Seoul Korea

**Keywords:** alopecia, androgenetic alopecia, female pattern hair loss, hair transplant, scalp micropigmentation, tattooing

## Abstract

**Background:**

Hair transplant (HT) is the standard treatment for female pattern hair loss (FPHL), but scalp micropigmentation (SMP) is an alternative. Currently, there are no criteria to help choose between HT and SMP.

**Methods:**

Forty female patients with FPHL who had successfully undergone HT and SMP, were reviewed to identify factors that might help decide between treatments. Hair density (HD) and hair caliber were measured at the part line and mid‐occipital region by a Folliscope. The sensitivity and specificity of HT and SMP were determined by area under the curve (AUC) and receiver operating characteristic curve.

**Results:**

Patients were divided into HT (*n* = 23) and SMP (*n* = 17) groups. The follicular unit density (FUD) (HT: 62.06 ± 4.8551/cm^2^; SMP: 66.59 ± 3.4971/cm^2^) and HD (HT: 96.16 ± 16.6954/cm^2^; SMP: 116.08 ± 17.0520/cm^2^) were significantly different (*p* < 0.01) between groups. The AUC for FUD was 77.6% with a cutoff value of 66.83 and 87.0% (1–0.412) sensitivity. The AUC for HD was 82.4% with a cutoff value of 96.17 and 69.6% (1–0.118) sensitivity.

**Conclusions:**

HD was the most important factor when deciding between HT surgery and SMP. SMP is recommended when the HD is ≥104.6 hairs/cm^2^ and HT surgery is strongly recommended when the HD is ≤96.17 hairs/cm^2^.

## 1 INTRODUCTION

Female pattern hair loss (FPHL) is a type of androgenetic alopecia different from male pattern hair loss. FPHL, the most common cause of hair loss in women, can be categorized as a type of non‐scarring alopecia, which can also occur infrequently in men.^1^, ^2^, ^3^ FPHL can occur in individuals of all ages and it has a relatively high prevalence, which has a significant impact on the psychological, social, and mental well‐being of patients. Women with FPHL often experience a severe degree of stress related to quality of life and may develop feelings of inferiority in terms of outward appearance. FPHL manifests as a decrease in hair caliber and density without hairline receding as generally seen in male patients and it affects the entire scalp, excluding the foremost 3–4 cm of the anterior hairline. Patients with FPHL often express two main concerns: thinning hair with reduced volume and increased scalp visibility.

Treatment options for FPHL include topical minoxidil, low‐dose oral minoxidil, platelet‐rich plasma, finasteride, or dutasteride, which are limited to post‐menopausal women, or lifestyle modification, hair transplant surgery, and scalp micropigmentation (SMP).^4^, ^5^, ^6^

Hair transplant surgery has long been considered the standard treatment for FPHL.^7^ However, subjective satisfaction tends to be lower than that in male patients because FPHL affects the entire scalp. Moreover, many cases involve thin and low‐density donor hair, further limiting the surgical outcome. Therefore, targeting selective areas such as the part line with increased scalp visibility may help to achieve favorable results.

The use of SMP for FPHL was first reported by Park.^8^ Since then, it has been accepted as an effective treatment for FPHL, especially for camouflaging scalp visibility rather than increasing hair volume. SMP helps conceal balding areas using tattoo techniques to mimic natural hair follicles and the small fine SMP dots on the scalp reduce the scalp see‐through appearance.

It is a dilemma whether to perform hair transplant surgery or SMP when patients with FPHL visit a hair transplant clinic. To the best of our knowledge, no reports have discussed the criteria for choosing between hair transplantation and SMP or the sequence of treatment plans. Therefore, we conducted a retrospective study of FPHL patients who had successfully undergone SMP or hair transplant surgery. This study investigated the relationship between various factors including symptoms, hair caliber and density, and the choice of therapeutic regimen. Here, we report the study outcomes and provide a literature review.

## 2 MATERIALS AND METHODS

The study included 40 patients with FPHL with a successful outcome after undergoing hair transplant surgery or SMP at our clinic. The study targeted patients who had high subjective satisfaction and excellent improvement by global photographic assessment 6 months after hair transplant surgery or SMP.

All patients were female. Medical charts and photographs were reviewed retrospectively. The average age of patients was 50.2 ± 12.4 years, ranging from 27 to 65 years. All patients were East Asian with black hair and most of them had Fitzpatrick type III skin.

Those with a history of dermatologic conditions possibly affecting the scalp, facial aesthetic surgery involving incisions into the scalp (forehead reduction, facial lifting surgery), and those with a history of previous hair transplant surgery or SMP were excluded from the study. Preoperative evaluation and examinations were performed by three expert hair surgeons with over 10 years of experience in both procedures.

This study was conducted in accordance with the Declaration of Helsinki for research in human subjects. All patients gave written consent for surgery and the use of photographs.

### 2.1 Preoperative consultation and evaluation

1. Family history, medication history, medical history, and surgical history were examined.
2. Subjective chief complaints were confirmed through detailed and thorough consultations.
3. Objective physical examinations, performed by a physician, checked whether the contour of the skull was visible in the frontal view. The phenomenon of the skull contour exposed through hair, also known as mountain ridge sign (MRS) was examined, rated, and categorized into the following three types: MRS negative, MRS slight positive, and MRS significantly positive (Figure 1).
4. Hair transplant surgery or SMP was decided upon after patient interview and physical examinations.
5. Using a Folliscope™ (LeadM, Seoul, Korea), the hair density (HD) and caliber were measured at three points on the part line and the mid‐occipital region of the occipital donor area, and the coverage value (CV) was calculated (Figure 2) in follicular units per square centimeter multiplied by the number of hairs per follicle and average hair diameter. In measuring the three points on the part line, the center point was initially determined as the midpoint between the frontal hairline and the parietal whorl, then two additional points spaced 3 cm anterior and posterior to the midpoint along the part line were measured, altogether forming the three measurement points.
6. The treatment plan determined by the physician was used to divide the patients into two groups: a hair transplant group (HT group) and SMP group, and the HD, thickness, CV, and objective chief complaints based on physical examination were retrospectively analyzed for correlation with the chosen treatment plan. Statistical analyses were performed using Win SPSS version 26.0 software.

**FIGURE 1:**
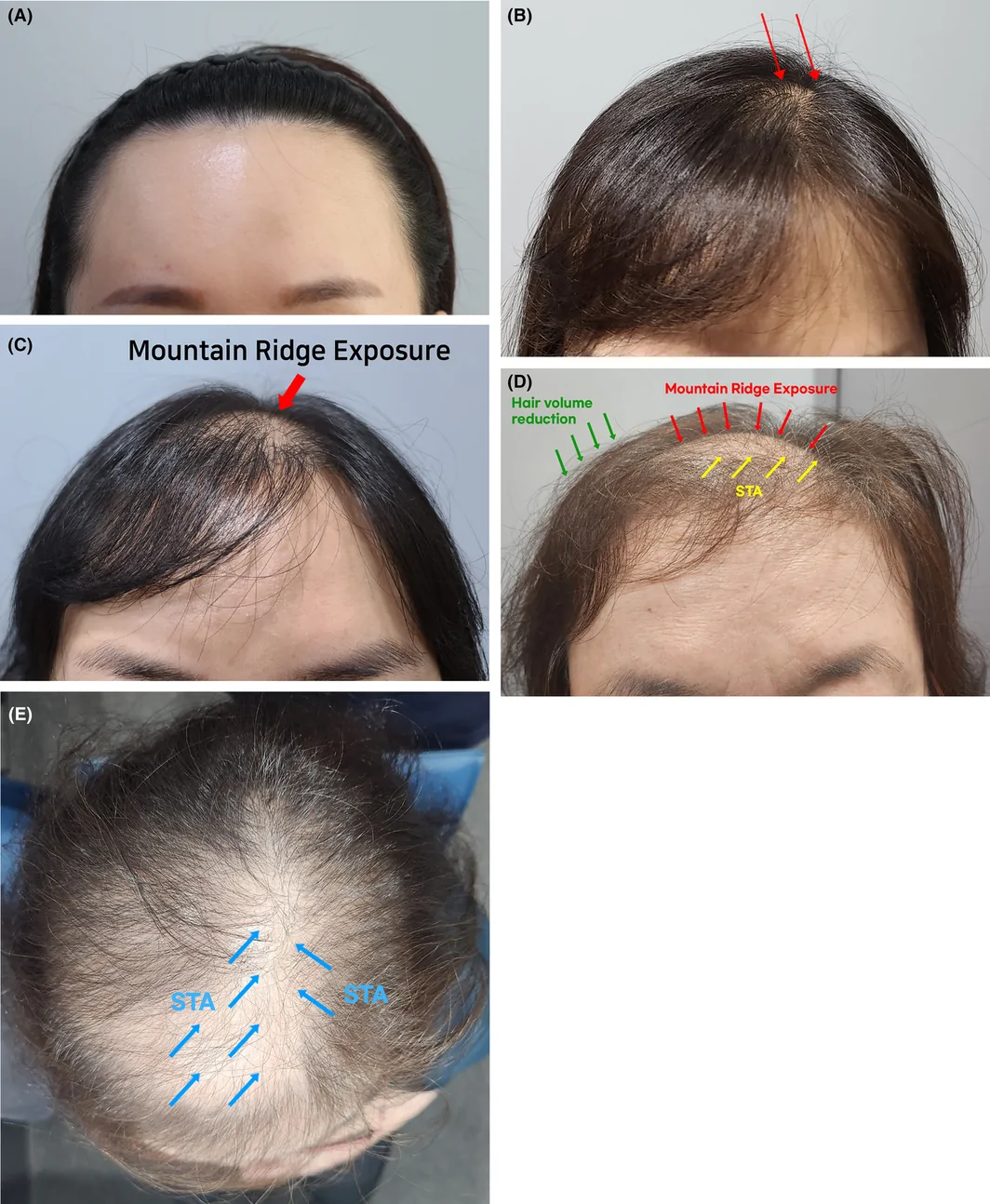
Mountain ridge sign. (A) Negative mountain ridge sign with the skull contour invisible. (B) Slightly positive mountain ridge sign. (C) Significantly positive mountain ridge sign. (D) Frontal view of a female patient with very advanced stage female pattern hair loss (FPHL). (E) Top view of a female patient with very advanced stage FPHL. STA, see‐through appearance.

**FIGURE 2:**
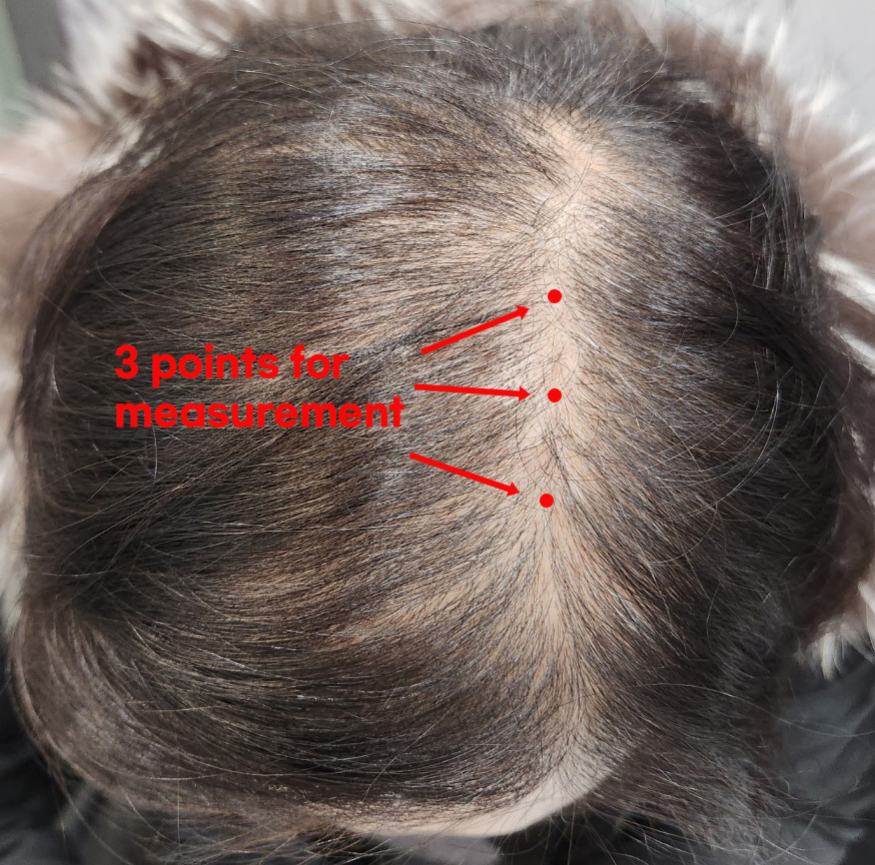
Three measurement points along the part line.

## 3 RESULTS

Of the 40 patients in this study, 23 underwent hair transplant surgery and 17 underwent SMP. Of these, 27.5% (*n* = 11) of patients were MRS slight positive, 57.5% (*n* = 23) were MRS negative, and 15% (*n* = 6) were MRS significantly positive.

### 3.1 Mountain ridge sign

In the HT group, MRS was slightly positive in 17.4% of patients (*n* = 4), negative in 56.5% (*n* = 13), and significantly positive in 26.1% (*n* = 6). In the SMP group, MRS was slightly positive in 41.2% (*n* = 7), negative in 58.8% (*n* = 10), and significantly positive in 0.0% (*n* = 0). Fisher's exact test revealed statistically significant associations with a *p* value <0.05 (Table 1).

**TABLE 1:** Comparison of mountain ridge signs between the HT and SMP groups.

		Treatment method		Total	*χ* ^2^ (*p* value)
		HT group	SMP group		
Mountain ridge sign	Slightly positive	4 (17.4)	7 (41.2)	11 (27.5)	6.425 (0.043*)
	Negative	13 (56.5)	10 (58.8)	23 (57.5)	
	Positive	6 (26.1)	0 (0.0)	6 (15.0)	
Total		23 (100.0)	17 (100.0)	40 (100.0)	

*Note*: Statistics were analyzed by Fisher's exact test (percentile).

Abbreviations: HT, hair transplant; SMP, scalp micropigmentation.

* *p* < 0.05.

### 3.2 Follicular unit density and hair density of the part line

Follicular unit density (FUD) measured at the part line was 62.06 ± 4.8551/cm^2^ in the HT group and 66.59 ± 3.4971/cm^2^ in the SMP group, which was significantly different (99.0% confidence level; *p* < 0.01). HD in the part line was 96.16 ± 16.6954/cm^2^ in the HT group and 116.08 ± 17.0520/cm^2^ in the SMP group, which was also significantly different (99.0% confidence level; *p* < 0.01) (Table 2).

**TABLE 2:** Comparison of FUD and HD between the HT and SMP groups.

Treatment		*N*	M	SD	*t*	*p* value
Part line FUD	HT group	23	62.06	4.8551	−3.267	0.002**
	SMP group	17	66.59	3.4971		
Part line HD	HT group	23	96.16	16.6954	−3.697	0.001**
	SMP group	17	116.08	17.0520		

*Note*: Statistics were analyzed by *t*‐test (mean (M) ± standard deviation (SD)).

Abbreviation: FUD, follicular unit density; *N*, number; SMP, scalp micropigmentation.

** *p* < 0.01.

### 3.3 Hair thickness in the part line

Hair thickness in the part line was 91.71 ± 5.0164/cm^2^ in the HT group and 94.02 ± 5.6525/cm^2^ in the SMP group, which did not reach statistical significance (Table 3).

**TABLE 3:** Comparison of hair thickness in the part line between the HT and SMP groups.

Treat		*N*	M	SD	*t*	*p* value
Hair thickness in the part line	HT group	23	91.71	5.0164	−1.364	0.181
	SMP group	17	94.02	5.6525		

*Note*: Statistics were analyzed by *t*‐test (mean (M) ± standard deviation (SD)).

Abbreviations: HT, hair transplant; *N*, number; SMP, scalp micropigmentation.

### 3.4 CV in the part line

The CV was 9.2 ± 0.502 in the HT group and 9.4 ± 0.565 in the SMP group, which did not reach statistical significance (Table 4).

**TABLE 4:** Comparison of the CV in the part line between the HT and SMP groups.

Treat		*N*	M	SD	*t*	*p* value
Part line CV	HT group	23	9.2	0.502	−1.364	0.181
	SMP group	17	9.4	0.565		

*Note*: Statistics were analyzed by *t*‐test (mean (M) ± standard deviation (SD)).

Abbreviations: CV, coverage value; HT, hair transplant; *N*, number; SMP, scalp micropigmentation.

### 3.5 Follicular density and HD in the occipital donor area

Follicular density in the occipital donor area was 72.22 ± 3.9423 in the HT group and 73.65 ± 3.6218 in the SMP group, which did not reach statistical significance at a 95% confidence level. HD in the occipital donor area was 142.43 ± 16.4367 in the HT group and 149.94 ± 7.8698 in the SMP group, which did not reach statistical significance at a 95% confidence level (Table 5).

**TABLE 5:** Comparison of the FUD and HD between the HT and SMP groups.

Treat		*N*	M	SD	*t*	*p* value
Occipital FUD	HT group	23	72.22	3.9423	−1.173	0.248
	SMP group	17	73.65	3.6218		
Occipital HD	HT group	23	142.43	16.4367	−1.913	0.064
	SMP group	17	149.94	7.8698		

*Note*: Statistics were analyzed by *t*‐test (mean (M) ± standard deviation (SD)).

Abbreviations: FUD, follicular unit density; HD, hair density; HT, hair transplant; SMP, scalp micropigmentation; *N*, number.

### 3.6 Hair thickness in the occipital donor area

Hair thickness in the occipital donor area was 102.57 ± 5.1153 in the HT group and 103.82 ± 5.0029 in the SMP group, which did not reach statistical significance (Table 6).

**TABLE 6:** Comparison of hair thickness in the occipital donor area between the HT and SMP groups.

Treat		*N*	M	SD	*t*	*p* value
Occipital hair thickness	HT group	23	102.57	5.1153	−0.776	0.442
	SMP group	17	103.82	5.0029		

*Note*: Statistics were analyzed by *t*‐test (mean (M) ± standard deviation (SD)).

Abbreviations: FUD, follicular unit density; HD, hair density; HT, hair transplant; *N*, number; SMP, scalp micropigmentation.

### 3.7 Coverage value in the occipital donor area

In the occipital donor area, the CV was 10.3 ± 0.512 in the HT group and 10.4 ± 0.500 in the SMP group, which did not reach statistical significance (Table 7).

**TABLE 7:** Comparison of CV of the occipital donor area between the HT and SMP groups.

Treat		*N*	M	SD	*t*	*p*‐value
Occipital CV	HT group	23	10.3	0.512	−0.776	0.442
	SMP group	17	10.4	0.500		

*Note*: Statistics were analyzed by *t*‐test (mean (M) ± standard deviation (SD)).

Abbreviations: CV, coverage value; HT, hair transplant; *N*, number; SMP, scalp micropigmentation.

In this study, the only variables that were statistically significant between the groups were the FUD and HD. Therefore, we examined the sensitivity and specificity of the two methods using area under the curve (AUC) and receiver operating characteristic (ROC) curves. The ROC curve depicts the relationship between sensitivity and specificity through a graph, and the AUC represents the area under this ROC curve. The closer the value of this area is to 1, the better the classification performance of the model.

### 3.8 Sensitivity and specificity of the FUD and HD in the part line between the two groups

The AUC for FUD in the part line was 77.6% (*p* < 0.01) with a cutoff value of 66.83 and 87.0% (1–0.412) sensitivity. Hair transplant is recommended when the FUD is 66.83 or lower. The AUC for HD was 82.4% (*p* < 0.01) with a cutoff value of 96.17 and 69.6% (1–0.118) sensitivity; therefore, hair transplant surgery is recommended when the FUD is 66.83 or lower and the HD is 96.17 or lower (Table 8).

**TABLE 8:** Sensitivity and specificity of the part line FUD and HD between the HT and SMP groups.

	AUC	SE	*p* value	cutoff value	sensitivity	specificity
Part line FUD	0.776	0.074	0.003**	66.8333	0.870	0.588
Part line HD	0.824	0.065	0.001**	96.1667	0.696	0.882

Abbreviations: AUC, area under the curve; SE, standard error.

** *p* < 0.01.

### 3.9 Comparative analysis between the two groups by age

The mean age of the HT group was 57.5 years, while the SMP group had a mean age of 42.5 years. Fisher's exact test was conducted to investigate whether there was a difference in treatment choice based on age distribution, but the results indicated that there was no statistically significant difference between the two groups by age, with *p* > 0.05 (Table 9).

**TABLE 9:** Comparison between the two groups by age group.

	Treatment method		Total	*χ* ^2^ (*p* value)
	SMP group	HT group		
Twenties	2 (66.7)	1 (33.3)	3 (100.0)	6.068 (0.079)
Thirties	2 (40.0)	3 (60.0)	5 (100.0)	
Forties	7 (70.0)	3 (30.0)	10 (100.0)	
Fifties	3 (33.3)	6 (66.7)	9 (100.0)	
Sixties and more	3 (23.1)	10 (76.9)	13 (100.0)	
Total	17 (42.5)	23 (57.5)	40 (100.0)	

*Note*: Statistics were analyzed by Fisher's exact test. (percentile).

Abbreviations: HT, hair transplant; SMP, scalp micropigmentation.

## 4 DISCUSSION

The primary symptoms of patients with FPHL are scalp visibility and hair volume reduction.^9^ Scalp see‐through appearance is noticeable when viewed from the front and above. However, volume reduction is most apparent when seen in the frontal view because hair thinning or decreased HD leads to a sunken appearance with little volume along with scalp visibility. When viewed from the top, the symptom of scalp see‐through appearance is dominant over that of volume reduction (Figure 3).

**FIGURE 3:**
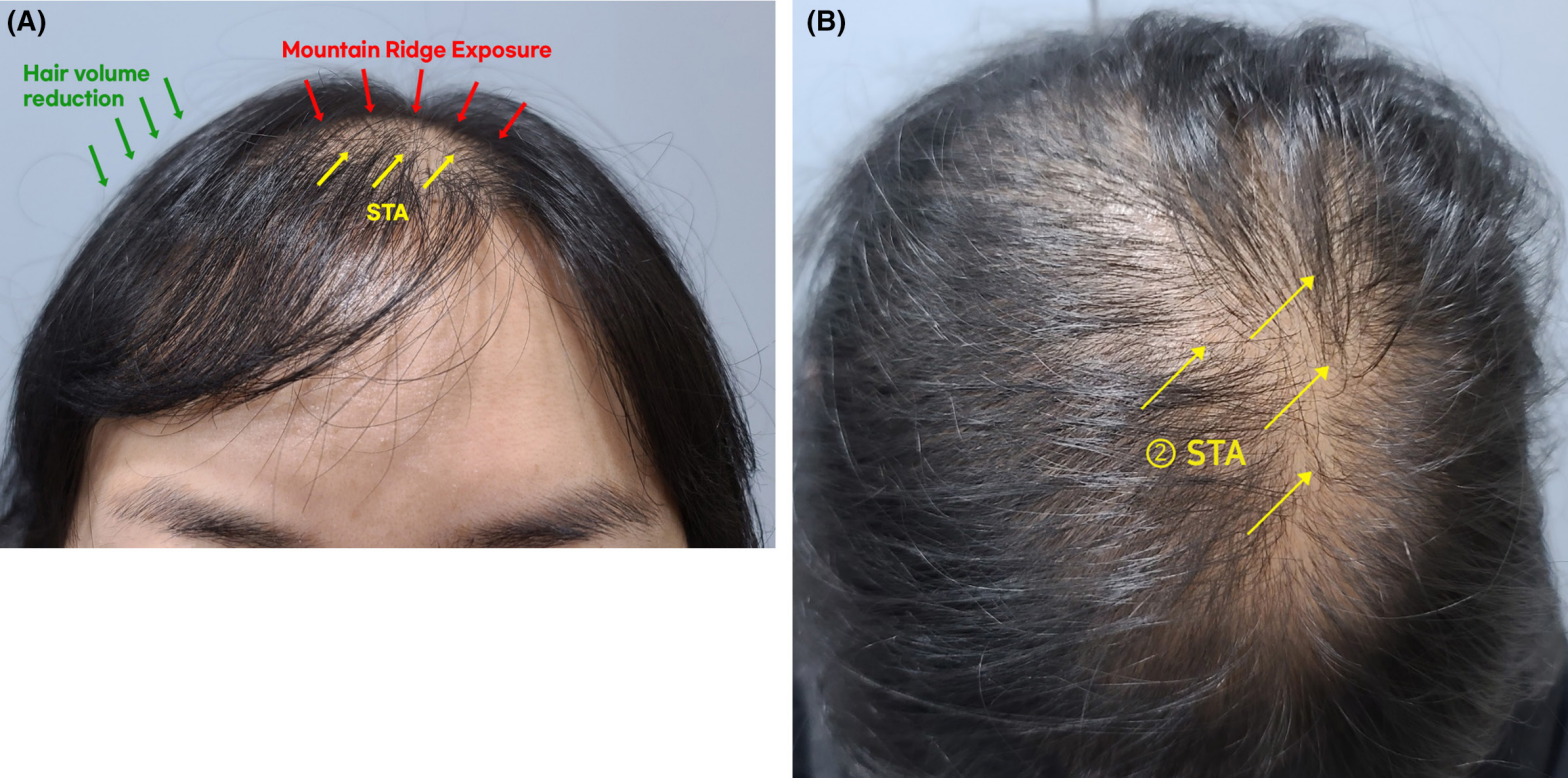
Main symptoms of patients with female pattern hair loss (FPHL). (A). Viewed from the front: ① hair volume reduction, ② see‐through appearance (STA), ③ mountain ridge sign (+). (B) Viewed from above: STA.

Scalp visibility is usually the major complaint of patients with FPHL, which is influenced by the degree of light‐colored scalp visible through the hair. Park reported that scalp visibility is determined by the contrast in color between the hair and scalp because more bright spectrum light is reflected from the scalp and into the visual system.^10^ Therefore, the SMP method reduces the reflection of light by introducing small dark pigment to a light‐colored scalp (Figure 4).^11^

**FIGURE 4:**
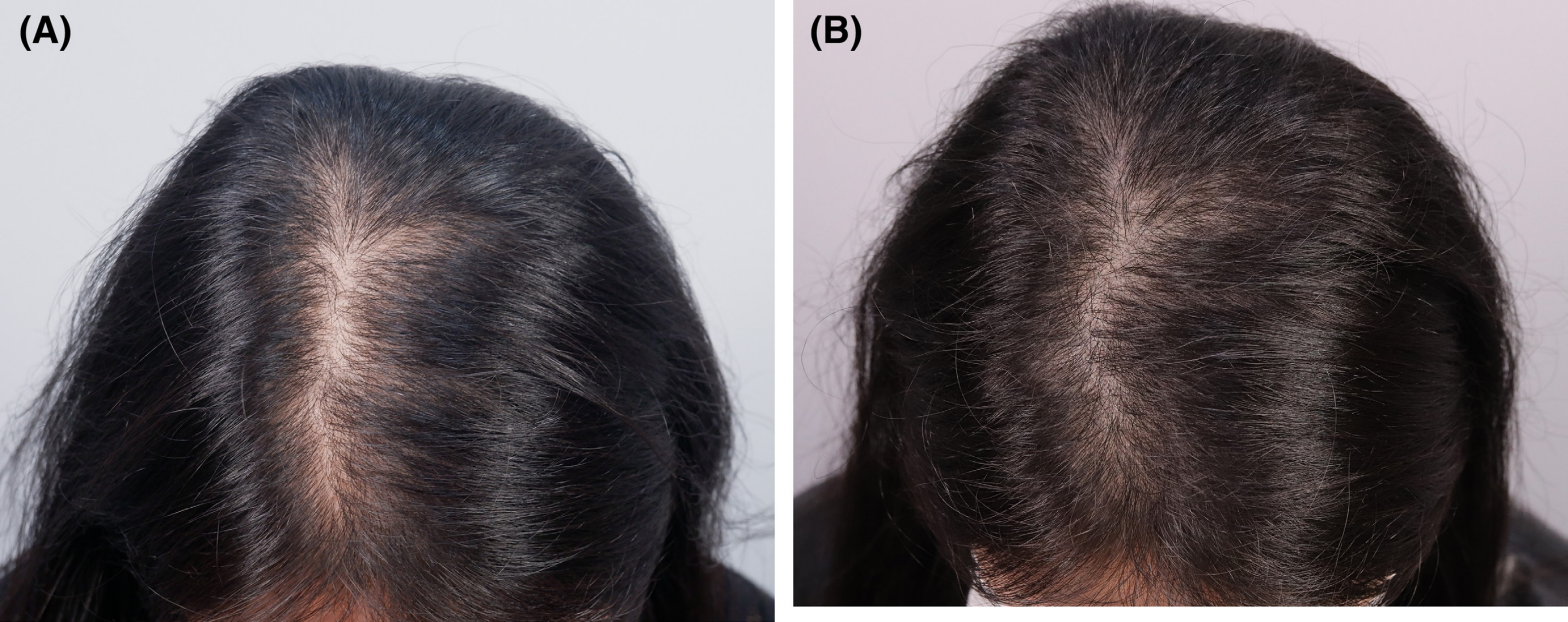
A 56‐year‐old patient with female pattern hair loss (FPHL) after scalp micropigmentation (SMP). (A) Before SMP. (B) Six months after SMP: SMP was applied to an 8‐cm wide, 15‐cm long area at the part line. Scalp see‐through appearance has been ameliorated to a great degree.

The loss of hair volume, which causes most stress to women with FPHL, is noticeable when seen from the front and is related to a decrease in HD. Such a reduction in volume cannot be ameliorated by SMP. Although SMP may give the illusion of slightly increased volume, the ultimate solution to restoring hair volume is hair transplant surgery.

Generally, scalp visibility and volume loss are the main complaints of patients with FPHL. However, the contour of the skull seen through hair strands in those with advanced alopecia when viewed from the front is mainly observed during physical examination. This symptom becomes noticeable in patients where the FPHL has progressed significantly and is similar to looking at a distant mountain. The ridge of the mountain is not visible when it is covered with trees, but the ridge becomes exposed and visible as the mountain becomes barren. Therefore, we named the phenomenon the "mountain ridge sign." SMP cannot solve the issues when MRS is positive (the skull's contour is visible from the front). Therefore, we consider MRS positive is a strong indicator for hair transplant surgery.

Figure 5 shows a conceptual diagram of the mountain ridge theory. In Figure 5A, the outline of the skull is the mountain ridge, and the trees represent hair. Figure 5B represents early‐stage hair loss with a slightly reduced number of trees. In Figure 5C, alopecia has progressed to a higher stage with more trees missing. At this point, the skull contour has been exposed, and the MRS is positive. Figure 5D delineates how the mountain ridge sign remains, even after SMP. Figure 5E shows hair transplant surgery is required at this stage to resolve such a phenomenon. Figure 6 shows an improved state in an MRS positive patient after hair transplant surgery. Hair loss can be simply described as a decrease in hair thickness and/or HD. Reduced HD is a criterion for considering hair transplantation surgery (Figure 7). If the main cause of scalp visibility is deceased hair caliber rather than decreased HD, then hair transplant surgery may do more harm than good by causing damage to adjacent hair follicles or worsening the situation. Thus, SMP may be a more suitable choice in this case (Figure 8).^12^

**FIGURE 5:**
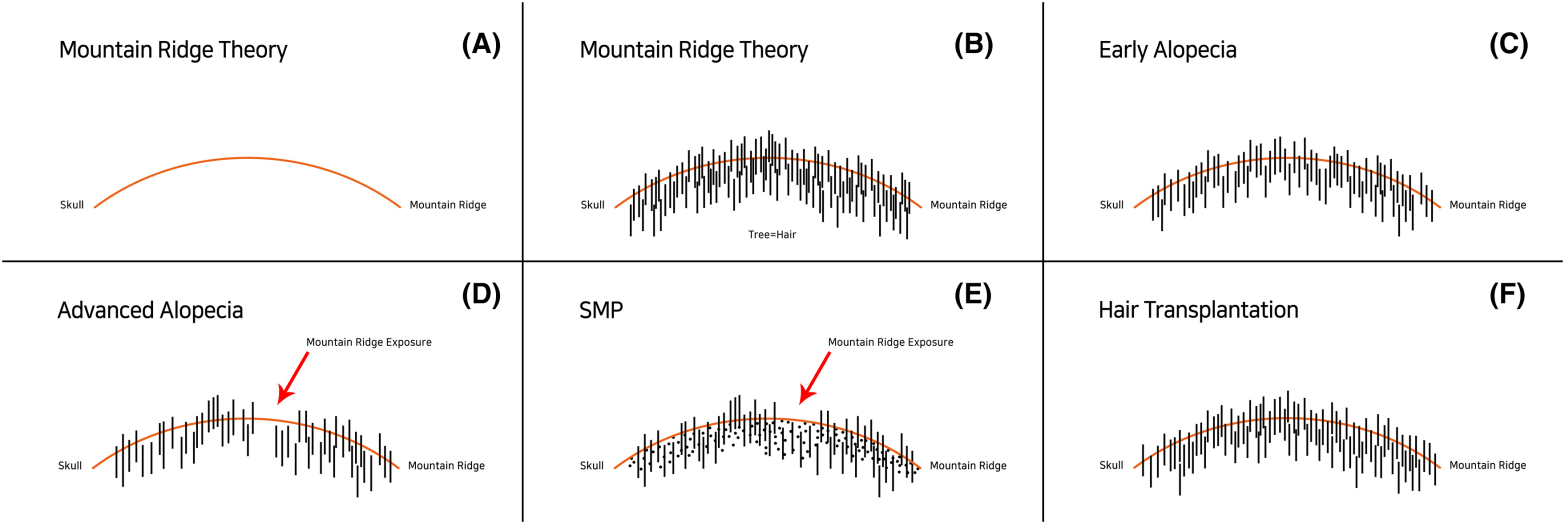
A conceptual diagram of the mountain ridge theory. (A) The outline of the skull is referred to as a mountain ridge. (B) Without alopecia (trees = hairs). (C) Early‐stage alopecia. (D) Advanced stage alopecia positive for mountain ridge sign. (E) Mountain ridge sign sustained even with scalp micropigmentation (SMP). (F) Mountain ridge sign resolved only after hair transplant surgery.

**FIGURE 6:**
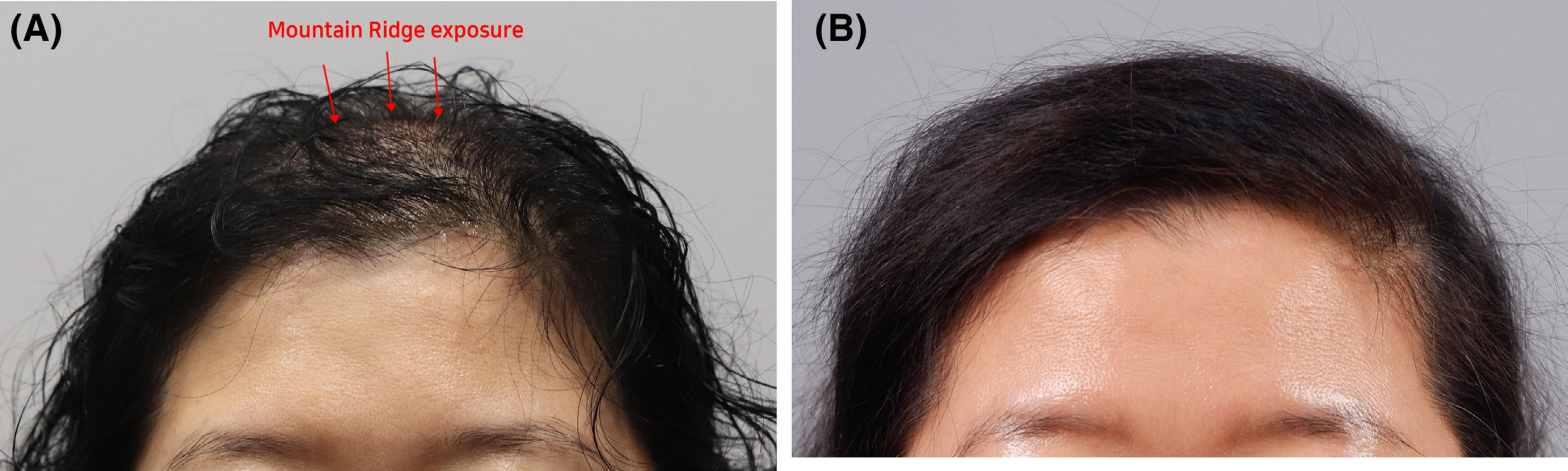
A 60‐year‐old female patient positive for mountain ridge sign (MRS). (A) MRS (+) prior to hair transplant surgery. (B) One year after hair transplant surgery: the MRS is greatly reduced.

**FIGURE 7:**
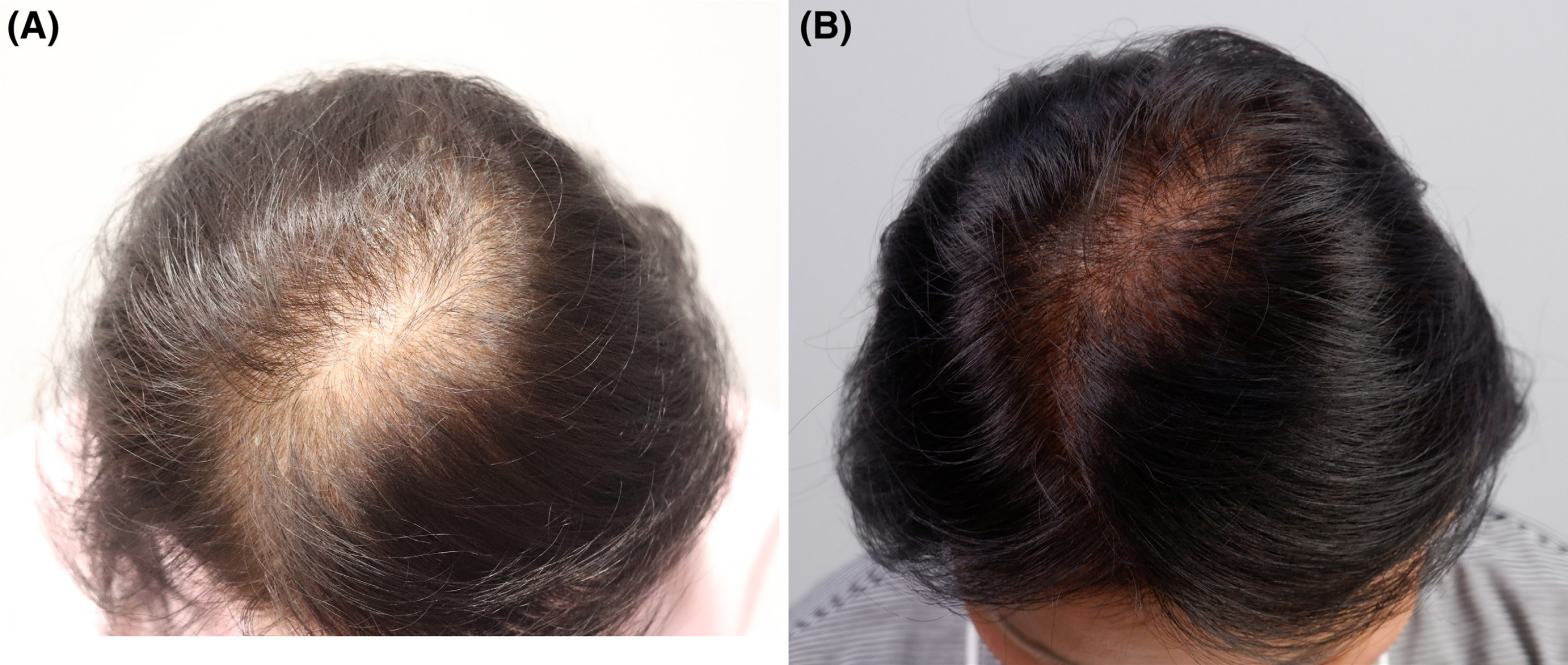
A 47‐year‐old female patient with hair transplant surgery of 1650 FUs by Follicular unit excision (FUE). (A) Prior to hair transplant surgery. (B) One year after hair transplant surgery.

**FIGURE 8:**
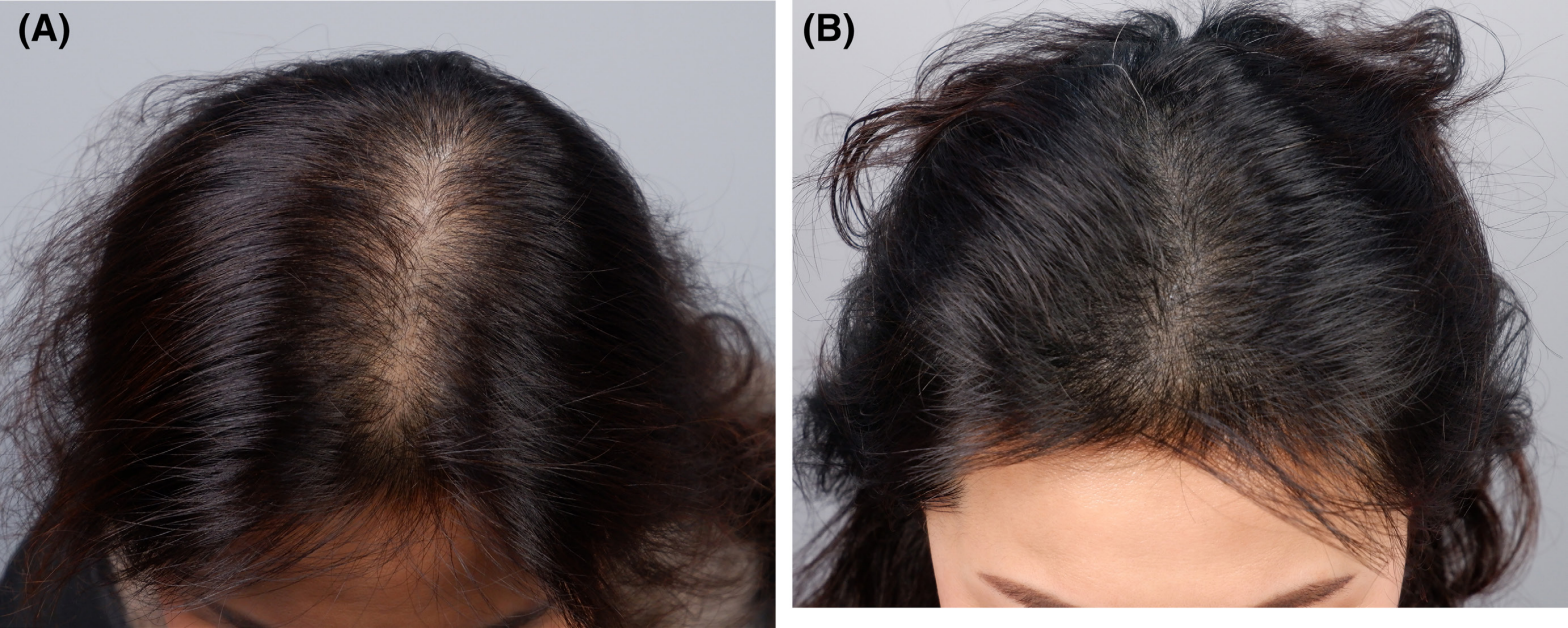
A 48‐year‐old female patient with female pattern hair loss (FPHL) with SMP in an area of 11 × 15 cm. (A) Prior to scalp micropigmentation (SMP). (B) Ten months after SMP.

In this study, MRS in the HT group was slightly positive in 17.4% of patients (*n* = 4), negative in 56.5% (*n* = 13), and significantly positive in 26.1% (*n* = 6). In the SMP group, it was slightly positive in 41.2% (*n* = 7), negative in 58.8% (*n* = 10), and significantly positive in 0.0% (*n* = 0). This indicates that the MRS is a strong indicator for hair transplant surgery.

When comparing the average values, the FUD and HD were 63.2 FU/cm^2^ and 93 hairs/cm^2^ in those with significantly positive MRS, 64.3 FU/cm^2^ and 99.9 hairs/cm^2^ when slightly positive, and 64 FU/cm^2^ and 109.6 hairs/cm^2^ when negative. Thus, the MRS is closely related to Hd. All cases with prominent MRS were categorized into the HT group, demonstrating the MRS is a strong indicator for hair transplant surgery.

Our retrospective study demonstrated the only variables that were significantly different between the HT group and SMP group were the FUD and HD of the scalp affected by alopecia. There were no statistically significant differences between the two groups in terms of hair thickness, CV, FUD, HD of the occipital donor area, or hair thickness.

A closer examination of the two variables with statistically significant differences between the two groups revealed that the average HD was 96.16 ± 16.6954 in the HT group, and the average HD was 116.08 ± 17.0520 in the SMP group, with a crossover point of 104.6 between the two groups.

In summary, the critical deciding factor between hair transplant surgery and SMP is HD in the alopecic area. In this study, surgery was recommended when the HD was lower than 104.6 hairs/cm^2^ as a decrease in HD outweighed reduced hair caliber. Conversely, SMP was recommended for patients with HD equal to or higher than 104.6 hairs/cm^2^.

The AUC analysis of the balding area demonstrated that HD lower than 104.6 hairs/cm^2^, with a cutoff value of 96.17, had the highest sensitivity and specificity values. Therefore, surgery is strongly recommended when the HD is 96.17 hairs/cm^2^ or lower.

It is essential to select the most adequate treatment plan for FPHL. As shown, more satisfying results may follow when using the cutoff values presented in this study when decision‐making between hair transplant surgery and SMP.

## 5 STUDY LIMITATIONS

This study was primarily focused on Asian females. Scalp visibility depends on the color contrast between the hair and scalp. Therefore, the results may not be duplicable in African Americans or Caucasians with lower color contrast, possibly leading to less subjective symptoms. There are also differences in HD, caliber, color, and curliness in patients of different ethnic backgrounds. Therefore, the study results may not be directly applicable to other ethnic groups, and further research should follow in the future.

## 6 CONCLUSION

In summary, HD is the most important factor for deciding between hair transplant surgery and SMP. SMP is recommended for patients with HD equal to or higher than 104.6 hairs/cm^2^. Cases with a density of 104.6 hairs/cm^2^ or less are advised to undergo hair transplantation, whereas those with 96.17 hairs/cm^2^ or less are strongly recommended to undergo hair transplant surgery. Also, when MRS is significantly positive, it becomes a strong indicator for hair transplant surgery compared to SMP.

## FUNDING INFORMATION

None were declared. No funding was received for this article.

## ETHICS STATEMENT

The study was performed in accordance with the principles of the Declaration of Helsinki.

## Data Availability

In addition to photographic evidence showing interval changes that appear in the manuscript, complete medical records of the presented cases are stored in the treating clinic, in line with the standard of care.
